# Effects of N-acetylcysteine on the energy status and antioxidant capacity in heart and liver of cold-stressed broilers

**DOI:** 10.5713/ajas.19.0542

**Published:** 2019-11-12

**Authors:** Chengcheng Li, Meng Peng, Man Liao, Shuangshuang Guo, Yongqing Hou, Binying Ding, Tao Wu, Dan Yi

**Affiliations:** 1 Hubei International Scientific and Technological Cooperation Base of Animal Nutrition and Gut Health, Wuhan Polytechnic University, Wuhan 430023, China

**Keywords:** Antioxidative Capacity, Broiler, Cold Stress, Energy Status, N-acetylcysteine

## Abstract

**Objective:**

Cold stress induces oxidative damage and impairs energy status of broilers. N-acetylcysteine (NAC) exhibits antioxidant properties and modulates energy metabolism of animals. This study was conducted to investigate the effects of NAC on energy status and antioxidant capacity of heart and liver in the cold-stressed broilers.

**Methods:**

The experiment consisted of 4 treatments in a 2×2 factorial arrangement with two diets (basal diet or plus 0.1% NAC) and two ambient temperatures (thermoneutral [conventional ambient temperature] or cold stress [10°C±1°C during days 15 to 42]).

**Results:**

No ascites were seen in cold-stressed broilers. NAC did not attenuate the impaired growth performance of stressed birds. However, NAC decreased plasma asparagine but increased aspartate levels in cold-stressed birds (p<0.05). NAC reduced hepatic adenosine triphosphate (ATP) but elevated adenosine diphosphate contents in unstressed birds (p< 0.05). The hepatic ratio of adenosine monophosphate (AMP) to ATP was increased in birds fed NAC (p<0.05). NAC decreased plasma malondialdehyde (MDA) level and cardiac total superoxide dismutase (T-SOD) activity in unstressed birds, but increased hepatic activities of T-SOD, catalase and glutathione peroxidase in stressed birds (p<0.05). NAC down-regulated hepatic AMP-activated protein kinase but up-regulated cardiac heme-oxigenase mRNA expression in stressed birds, and decreased expression of hepatic peroxisome proliferator-activated receptor coactivator-1α as well as hypoxia-inducible factor-1α in liver and heart of birds.

**Conclusion:**

Dietary NAC did not affect energy status but enhanced the hepatic antioxidant capacity by increasing the activities of antioxidant enzymes in cold-stressed broilers.

## INTRODUCTION

Cold stress is a great challenge for broilers during winter transportation. It is estimated that 20 million poultry deaths and an economic loss of 100 million can be attributed to the winter conditions and low temperatures in China [1]. In a low temperature, the metabolic rate of fast-growing broilers rises for heat production to maintain normal body temperature, leading to excess nutrient oxidation and oxygen deficiency [2]. Consequently, cardiac output increases to supply more oxygen for metabolism, which results in pathological alterations such as classic hematological changes and heart hypertrophy [3]. Long-standing pathologic hypertrophy will contribute to heart disorder and eventually increases the mortality and morbidity of chickens [4]. Of note, it has been well documented that exposure to low temperature induced the elevation of oxidative metabolism and heat production, accompanied by elevated reactive oxygen species (ROS) production and the oxidative stress in heart and liver [5]. In turn, oxidative damage further aggravates heart and liver dysfunction, which weakens the chickens. Additionally, cold stress has been also reported to impair the energy status by reducing cardiac energetic reserve, hepatic fatty acid oxidation and respiratory capacity [6].

To minimize the impairment caused by cold temperature, the techniques of nutritional manipulation are increasingly developed in broiler production, for example, dietary supplementation with vitamin C [7], copper-methionine [8], arginine and guanidinoacetic acid [2]. N-acetylcysteine (NAC) is a precursor of L-cysteine, which is utilized for the synthesis of reduced glutathione (GSH). A large body of evidence showed that NAC exhibited direct and indirect antioxidant properties via its free thiol group and the increase of cellular GSH concentration, respectively [9]. NAC has been in clinical practice for several decades and used for the treatment of cardiac injury, pulmonary disease, and cancer [10]. Intriguingly, in addition to its antioxidant actions, NAC may act as a vasodilator by facilitating the production and action of nitric oxide [11], implying that NAC plays a pivotal role in attenuating the constriction of blood vessels induced by cold temperature. Additionally, we have reported dietary NAC alleviated liver injury in a porcine model by improving the antioxidative capacity and energy metabolism [12]. Similarly, NAC also alleviates the toxic effects of aflatoxin in broilers by improving antioxidant capacity and energy metabolism [13]. Therefore, we hypothesized that NAC might benefit the hepatic and cardiac function of cold-stressed boilers by modulating the energy and antioxidant status. The present study was carried out to investigate the effects of NAC on the antioxidant capacity and energy status of liver and heart in broilers exposed to the low temperature, thereby providing the theoretical basis for the NAC application in poultry production.

## MATERIALS AND METHODS

### Animal care

The experimental procedures in the present study were approved by the Institutional Animal Care and Use Committee of Wuhan Polytechnic University (2016–1215).

### Birds, diets, and experimental design

Two hundred 1-day-old Cobb male chickens (45±0.50 g) were randomly assigned into 1 of 4 treatments, which contained 5 replicate pens with 10 birds per pen. The experiment consisted of 4 treatments in a 2×2 factorial arrangement with two diets (basal diet or plus 0.1% NAC) and two temperatures (thermoneutral or cold stress). The thermoneutral and cold stress treatments were in separate rooms equipped with air-conditioning systems. The corn-soybean meal based diet (Table 1) was formulated to meet National Research Council (1994)-recommended nutritional requirements, while 0.1% NAC (Sigma-Aldrich, St. Louis, MO, USA; Cat. A7250) was supplemented into the basal diet. NAC (powder) was well incorporated into the basal diet in one-batch mixing by using the quartering technique. The dosage of NAC was selected according to the study of Yi et al [13], who reported that 0.1% NAC was safe and increased the average daily feed intake (ADFI) and average daily gain (ADG) of broilers. Due to a negligible increase of 0.0084% nitrogen caused by NAC supplementation, we deemed it not necessary to use non-essential amino acids as an isonitrogenous control. The feed was manually supplied in feeders and water was easily available from the nipples of automatic drinking system. Both Feed and water were provided *ad libitum*. Lighting was kept at 23/1 light/dark cycle throughout the experiment.

The temperature of both rooms was set at 32°C for the first week and then reduced to 30°C in the second week. Then, temperature of one room (thermoneutral treatment) was gradually decreased by 2°C/week from d 15 until it reached 26°C±1°C by d 28 of age and remained constant thereafter. On the contrary, the temperature in the other room (cold stress treatment) was reduced to 10°C±1°C on d 15 of age and kept constant until the end of the experiment [2,7]. The experiment lasted for 42 days (6 weeks).

### Growth performance

Feed consumption and body weight (BW) for each replicate were recorded. The ADFI, ADG, feed conversion ratio (FCR) and final BW were calculated during days 1 to 14 and 15 to 42.

### Sample collection

On day 43 of age, 10 broilers per group (2 per replicate) were randomly selected and blood was collected from the wing veins. The plasma was then collected by centrifugation (3,000 r/min, 10 min) and stored at −20°C until analysis. Then, the chickens were euthanized by cervical dislocation. The thymus, spleen, bursa, heart, liver and lung were excised and weighed. The relative weights of organs (%) = organ weight / live weight × 100%. The samples from heart (right ventricle) and liver were collected, frozen in liquid nitrogen, and stored at −80°C for the determination of redox status and energy metabolism [14]. All samples were collected within 15 min.

### Measurement of plasma biochemical parameters

The concentrations of total protein (TP) and blood urea nitrogen (BUN), as well as the activities of glutamic-pyruvic transaminase (ALT), glutamic-pyruvic aminotransferase (AST), and alkaline phosphatase (ALP) in the plasma of broilers were determined by using a Hitachi 7020 automatic biochemical analyzer with WAKO chemical reagents (Wako Pure Chemical Industries, Ltd., Osaka, Japan) [15].

### Plasma amino acid analysis

Amino acids in the plasma were analyzed by an automatic amino acid analyzer (S433D, Sykam GmbH, Eresing, Germany) according to Xie et al [16] with minor modifications. Briefly, 1 mL of plasma and salicylsulfonic acid (2%) were mixed thoroughly and then placed in an ice bath for 15 min. After centrifuging (10,000 *g*) for 15 min, the supernatants were adjusted to pH 7.0 by adding a lithium hydroxide solution, and then mixed well. The liquid mixture was filtered through a 0.22 μm membrane and then analyzed for amino acid concentrations.

### Determination of energy status

The adenosine triphosphate (ATP), adenosine diphosphate (ADP), and adenosine monophosphate (AMP) levels in the liver and heart were determined by high-performance liquid chromatography (HPLC) method according to Yi et al [13]. Briefly, the samples (0.1 to 0.2 g) were homogenized with pre-cooled perchloric acid and then centrifuged to obtain supernatant. One milliliter of supernatant was put in a new tube and the same volume of potassium carbonate was added to adjust the pH to 7.4. This mixture was centrifuged to collect the supernatant. The contents of adenylate (AMP, ADP, and ATP) in the supernatant were determined by Waters Breeze HPLC system (Waters Corporation, Milford, MA, USA) with an analytical column (Waters XBridge C18; 5 μm, 4.6 mm×150 mm). The adenylate energy charges (AEC) and total adenine nucleotide (TAN) were calculated according to the flowing formulas: TAN = AMP+ADP+ATP; AEC = (ATP+ 0.5 ADP)/TAN. The contents of AMP, ADP, ATP, and TAN were expressed as microgram per gram of tissue wet weight.

### Determination of redox status

The measurement of redox status in heart and liver was conducted as Li et al [14] described. Briefly, the hepatic and cardiac samples (about 0.1 g) were homogenized with pre-cold saline and then centrifuged to collect the supernatant for the analysis of oxidative and anti-oxidative related parameters. The malondialdehyde (MDA) level and the activities of glutathione peroxidase (GSH-Px), catalase (CAT) and total superoxide dismutase (T-SOD) in plasma, liver and heart were determined by the commercially available kits (Nanjing Jiancheng Bioengineering Institute, Nanjing, China) according to the instructions of the manufacturer. The protein levels of hepatic and cardiac samples were analyzed using the Coomassie Brilliant Blue G-250 reagent with bovine serum albumin as a standard. Data of hepatic and cardiac samples were expressed based on protein contents.

### RNA isolation and quantitative real-time polymerase chain reaction

Total RNA isolation and quantitative real-time polymerase chain reaction (qRT-PCR) was conducted as described by Guo et al [17]. Briefly, total RNA of hepatic and cardiac samples was isolated using TRIzol Reagent protocol (Invitrogen, Carlsbad, CA, USA). The RNA was quantified by using the NanoDrop ND-1000A UV-VIS spectrophotometer (Thermo Scientific, Wilmington, DE, USA) and its purity was assessed by using 1% denatured agarose gel electrophoresis. One microgram of total RNA was reverse transcribed using a PrimeScript RT reagent kit with gDNA Eraser (Takara, Dalian, China) according to the manufacturer’s instruction. The cDNA was synthesized and stored at −20°C. The qRT-PCR assay was performed using the SYBR Premix Ex Taq (Takara, China) on an Applied Biosystems 7500 Fast Real-Time PCR System (Foster City, CA, USA). The primer sequences are listed in Table 2. To ensure the sensitivity and accuracy, both hepatic and cardiac samples were normalized internally using the average cycle threshold (Ct) of glyceraldehyde-3-phosphate dehydrogenase, which acted as an internal reference in each sample to avoid any artifacts from variation in the targeted genes. Data were calculated using the 2^−ΔΔCt^ method. Each biological sample was run in triplicate.

### Statistical analysis

The data were analyzed using the 2-factorial analysis of variance in SPSS17.0 (SPSS Inc., Chicago, IL, USA). The differences among means were evaluated by Turkey test when a significant interactive effect between two factors was observed. Additionally, the data of growth performance from d 1 to d 14 of age was analyzed by the Student’s unpaired *t*-test. Data were expressed as means and pooled SEM. Probability values ≤0.05 were taken to indicate significance.

## RESULTS

### Growth performance

The growth performance of broilers is summarized in Table 3. Broilers challenged with the cold stress had lower (p<0.05) ADFI, ADG, and BW than broilers under thermoneutral condition from day 15 to 42 of the age. Moreover, the FCR of broilers exposed to the cold stress was higher (p<0.05) than that of broilers in thermoneutral group. Additionally, dietary supplementation with NAC did not affect the growth performance of broilers under thermoneutral condition or cold stress.

### Relative weights of organs

As shown in Table 4, cold stress increased (p<0.05) the relative weights of heart and liver of broilers compared with the thermoneutral group. Moreover, there was a significant interaction between temperature and diet (p<0.05) for the relative weights of thymus and bursa. It was shown that diet supplemented with NAC alleviated the decrease in the relative weight of bursa in cold-stressed broilers. Interestingly, both NAC and cold stress decreased the relative weight of thymus.

### Plasma biochemical parameters

As seen in Table 5, the level of ALP in plasma of broilers exposed to the low temperature was higher (p<0.05) than that of broilers under thermoneutral condition. However, dietary NAC supplementation decreased (p<0.05) the plasma ALP levels of broilers in comparison with the basal diet group.

### Plasma amino acid profile

The profile of free amino acids in the plasma is shown in Table 6. Cold stress increased (p<0.05) the concentrations of isoleucine and lysine, methionine, and valine in plasma of broilers compared with broilers in the thermoneutral group. In contrast, cold stress decreased (p<0.05) the levels of serine, alanine, glutamine, proline, hydroxyproline and tryptophan in comparison with the thermoneutral group. NAC supplementation increased (p<0.05) the cystine and cysteine levels of broilers in comparison with the basal diet group. Moreover, there was a significant interaction (p<0.05) between temperature and diet in the concentrations of asparagine and aspartate, indicating that dietary supplement of NAC decreased the asparagine level, but increased the aspartate concentration in the plasma of broilers exposed to cold condition.

### Energy status

Compared with the thermoneutral group, cold stress increased (p<0.05) the levels of AMP and TAN, and AMP/ATP ratio in the liver, whereas decreased (p<0.05) the AEC level in the liver and ATP, AMP, and TAN concentrations in the heart of broilers (Table 7). Dietary NAC increased (p<0.05) the AMP/ATP ratio of broilers in comparison with the basal diet group. Additionally, there was a significant temperature × diet interaction (p<0.05) in hepatic ATP and ADP concentration. It was shown that both NAC and cold stress treatment increased ADP level, but decreased the ATP level in the liver.

### Redox status

As indicated in Table 8, broilers exposed to the low temperature had lower (p<0.05) activities of CAT and GSH-Px in the heart as well as CAT in the plasma, and higher MDA level in the heart than those in the thermoneutral group. Compared with the basal diet group, dietary supplementation of NAC decreased (p<0.05) the hepatic MDA concentration, whereas increased (p<0.05) the plasma GSH-Px activity of broilers. Additionally, significant temperature × diet interactions (p< 0.05) were observed in the activities of T-SOD in the liver and heart, CAT and GSH-Px in the liver, and MDA concentration in the plasma, indicating that dietary NAC elevated the T-SOD activity in the liver, and CAT and GSH-Px activities in the liver of cold-stressed broilers.

### Expression of genes related to redox and energy metabolism

**Figure 1:**
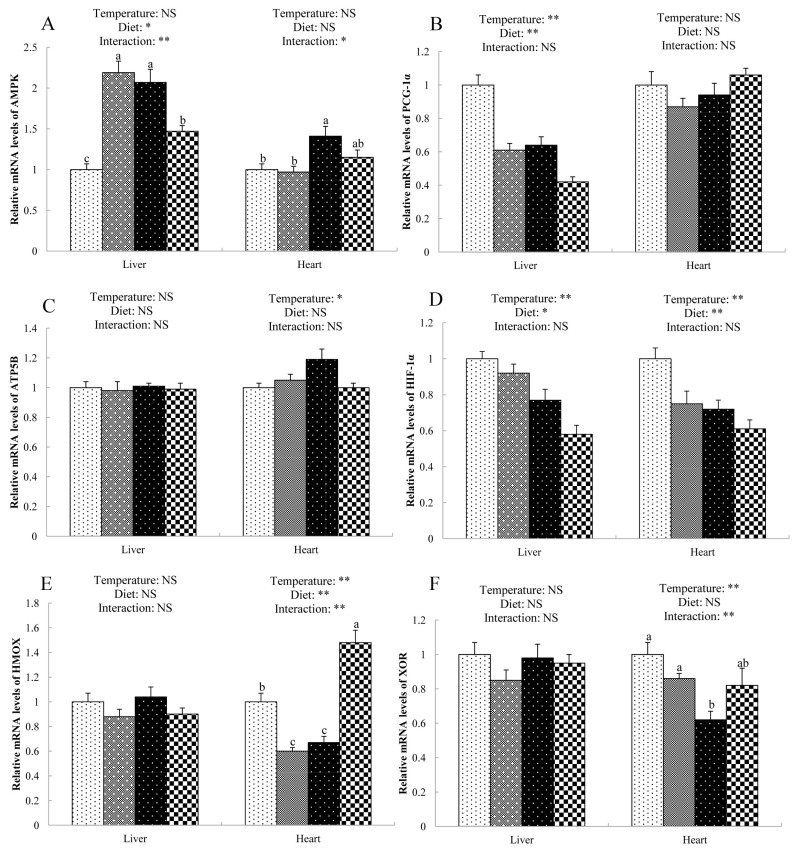
Gene expression in the liver and heart of cold-stressed broilers. (A) AMPK, AMP-activated protein kinase; (B) PGC-1α, peroxisome proliferator-activated receptor coactivator-1α; (C) ATP5B, ATP synthetase beta subunit; (D) HIF-1α, hypoxia-inducible factor-1α; (E) HMOX, heme-oxigenase; (F) XOR, xanthine oxidoreduct. ^a–c^ Means sharing different letters differ (p<0.05). * p<0.05, ** p<0.01, NS, no significance.

Both NAC and cold stress altered the gene expression in liver and heart of broilers (Figure 1). There were significant interactions (p<0.05) between diet and temperature in mRNA levels of AMP-activated protein kinase (AMPK, Figure 1A) in the liver and heart. NAC-fed broilers exposed to cold stress had lower mRNA level of AMPK in the liver and heart than those of cold-stressed birds fed basal diet. Compared with the thermoneutral group, cold stress induced the down-regulation (p<0.05) of peroxisome proliferator-activated receptor coactivator-1α (PGC-1α, Figure 1B) expression in the liver and hypoxia-inducible factor-1α (HIF-1α, Figure 1D) expression in liver and heart, and the up-regulation (p<0.05) of ATP synthetase beta subunit (ATP5B, Figure 1C) expression in the heart of broilers. Dietary supplementation of NAC decreased (p<0.05) the mRNA levels of PGC-1α and HIF-1α in the liver, and HIF-1α in the heart of broilers compared with broilers in the basal diet group. Diet and temperature showed interactive effects on the mRNA expression of heme-oxigenase (HMOX, Figure 1E) and xanthine oxidoreductase (XOR, Figure 1F) in the heart of broilers. NAC supplementation increased the HMOX mRNA abundance and alleviated the decrease of XOR expression in the heart of cold-stress broilers.

## DISCUSSION

Broilers are sensitive to temperature variations and their metabolic rate will rise to adapt to colder conditions [18], and thereby leading to the growth inhibition and even death [3]. Low ambient temperature is reported to induce hypoxia and ascites in broilers due to the increase of oxygen demands for both fast growth and heat production [3]. Several managements including nutritional and medicinal strategies are taken to minimize the loss in broilers due to the exposure to cold stress. Herein, we determined whether dietary NAC could improve hepatic and cardiac redox status and energy status in broilers under the low temperature based on the beneficial effects of NAC on heart, liver and intestinal of animals [11,12].

In the present study, the exposure to low temperature greatly impaired the growth performance of broilers. This was unsurprising, because heat production increased to maintain the normal body temperature of cold-stressed broilers and more nutrients were used for heat production [3]. As expected, cold stress increased the relative weights of heart and liver since low-temperature is an important inducer for hypertrophic growth of heart [4]. Moreover, cold stress decreased the relative weights of both thymus and bursa, which were responsible for the generation of T and B lymphocytes, respectively. It was reported that cold stress could suppress the immune responses of chickens [19]. Dietary NAC mitigated the decrease of bursa relative weight in cold-stressed broilers, which might further modulate the function of B lymphocytes that derived from bursa in chickens. In an *in vitro* experiment, NAC regulated the homeostasis of CD40-activated B lymphocytes isolated from human peripheral blood and showed immunomodulatory function with antioxidant-independent properties [20]. Therefore, NAC might play an immunomodulatory role in cold-stressed birds.

There are several indicators associated with hepatic diseases, such as the activities of ALT, AST, and ALP in blood [21]. Of note, blood ALP is related to hepatic disease caused by intra or extra hepatic cholestatis and some destruction of hepatic cell membrane [21]. Therefore, cold stress may result in liver dysfunction by observing that the activity of plasma ALP and relative weight of liver was increased in broilers exposed to low temperature in the present study. However, dietary NAC reduced the activity of plasma ALP in broilers under both thermoneutral and cold conditions, indicating that NAC could mitigate the liver injury. Additionally, the plasma TP and BUN levels were not affected by both treatments (diet and temperature), suggesting the protein metabolism was not influenced. Similar work in poultry is limited. However, in mammals, NAC was demonstrated to decrease BUN and improve kidney function through regulation of ammonia and nitrogen metabolism [22,23].

As mentioned above, cold stress could induce oxygen de ficiency (hypoxia), which would prevent the use of branched chain amino acids, including valine, leucine and isoleucine, in the mitochondrial electron transfer system of muscle [24]. Therefore, in the current study, the increased concentrations of valine and isoleucine in plasma might be due to the oxygen deficiency in muscle induced by low ambient temperature, which needs further investigation. Unlike the finding of Muratsubaki and Yamaki [24] that plasma lysine and methionine were not significantly affected by hypoxia, we found both were increased. These two amino acids might be modulated in a hypoxia-independent way under cold stress. Furthermore, cold stress reduced the levels of alanine, glutamine, proline, hydroxyproline, serine, and tryptophan in the plasma. This might be attributed to the liver injury (as mentioned above) and compromised intestinal integrity induced by cold stress [19], which decreased the availability of these amino acids. As ATP was required for proline synthesis [24], the reduced contents of ATP in heart and liver might also have contributed to the decrease of plasma proline level in broilers under cold condition. Additionally, NAC is a precursor of L-cysteine and undergoes extensive hepatic metabolism, resulting in increased levels of plasma cysteine, cystine, and GSH [9], which were consistent with the results of present study.

Cold stress as well as the fast growth augmented the met abolic rate of chickens, giving rise to enhanced oxidative metabolism, lipid peroxidation, and ROS production [25]. It is well known that ROS and related peroxides induced by cold stress lead to liver and heart injury. However, organisms can detoxify ROS using defense mechanisms (antioxidative enzymes) such as T-SOD, CAT, and GSH-Px [13]. These antioxidant enzymes cooperatively convert ROS into oxygen and water. In the current study, birds under cold stress exhibited the elevation of cardiac MDA content and reduction of GSH-Px, CAT, and T-SOD activities, indicating that low temperature resulted in an oxidative status. Meanwhile, cold stress induced the down-regulation of hepatic and cardiac HIF-1α as well as cardiac HMOX. Our results were in good agreement with the study of Osselaere et al [26], who also observed the down-regulation of hepatic HIF-1α and HMOX mRNA abundance in birds challenged with deoxynivalenol. HIF-1α is a core transcription factor regulating oxygen homeostasis and can activate the expression of many hypoxic reactive genes [27], while HMOX is associated with the protection against hepatocyte death [28]. It was stated that the up-regulation of HIF-1α usually happened in the first hours of oxygen deficiency and returned then to the basal level. In addition, cold stress also induced a down-regulation of cardiac XOR, an enzyme related to the synthesis of ROS [29]. The reasons for the down-regulation of XOR by cold stress are not clear. As an antioxidant, NAC enhanced the activity of antioxidative enzyme such as hepatic T-SOD, CAT, and GSH-Px, and reduced hepatic MDA level of cold-stressed broilers. The underlying mechanism whereby NAC exerted antioxidative function might be directly and indirectly associated with oxidants as reported by Yi et al [13]. Furthermore, NAC decreased the HIF-1α expression both in liver and heart but increased the HMOX and XOR mRNA levels in the heart of cold-stressed broilers, suggesting that NAC may regulate antioxidative capacity at a transcriptional level.

Except the elevations of triiodothyronine and leptin, cold stress increased the expression of uncoupling proteins, which uncoupled the respiration from ATP production to enhance body heat production and finally caused the reduction of ATP production [30]. Moreover, sustained hypoxia during cold exposure could result in a loss of cardiac energetic reserve, hepatic fatty acid oxidation and respiratory capacity [6]. Therefore, the present study observed the decrease of ATP concentration in liver and heart of cold-stressed broilers. Besides, cold stress increased the cardiac AMP/ATP ratio and reduced the hepatic AEC, indicating the impaired energy metabolism of birds. Unfortunately, dietary NAC did not show the improvements in energy metabolism, which may due to the insufficient experimental period. Nevertheless, NAC inhibited up-regulation of cardiac and hepatic AMPK, which serves as an energy sensor and is up-regulated by the elevating ratio of AMP/ATP [31]. ATP5B is a key enzyme in catalyzing the synthesis of ATP [32], and the up-regulation of cardiac ATP5B in cold-stressed broilers might be a feedback regulatory effects due to the decrease of ATP level in the heart. Another important gene, PGC-1α, which plays an important role in mitochondrial biogenesis and adaptive thermogenesis [33], was down-regulated in the liver of broilers with cold stress or NAC supplementation. The impaired liver function in cold-stressed birds might be responsible for the down-regulation of PGC-1α. However, the underlying mechanisms how dietary NAC decreased the hepatic PGC-1α expression need further investigation.

In conclusion, cold stress decreased the growth perfor mance, altered the plasma amino acids profile, induced the oxidative stress and hypertrophy in liver and heart, and impaired the hepatic and cardiac energy metabolism of broilers. Dietary supplementation with 0.1% NAC mitigated the oxidative stress by increasing the activities of antioxidant enzymes in the liver of cold-stressed broilers. The 0.1% NAC is recommended to use in the diets of cold-stressed birds.

## Figures and Tables

**Table 1:** Ingredients and chemical composition of the basal diet (air-dried basis)

Item	d 1 to 21	d 22 to 42
Ingredients (%)		
Maize, 8%1)	53.70	60.40
Soybean meal, 44%1)	28.20	24.90
Soybean oil	5.82	5.21
Corn gluten meal, 60%1)	5.00	5.00
Fishmeal, 65%1)	3.00	-
Dicalcium phosphate	1.80	1.74
Limestone	0.95	1.05
Choline chloride	0.20	0.20
Sodium chloride	0.18	0.31
L-lysine hydrochloride	-	0.14
DL-methionine	0.15	0.05
Premix2)	1.00	1.00
Nutritional composition		
Metabolizable energy (MJ/kg)3)	13.38	13.38
Crude protein (%)4)	23.0	20.0
Methionine (%)4)	0.54	0.39
Methionine+cysteine (%)4)	0.90	0.72
Lysine (%)4)	1.13	1.00
Threonine (%)4)	0.88	0.75
Calcium (%)3)	1.00	0.90
Total phosphorus (%)4)	0.75	0.65
Avaiable phosphorus (%)3)	0.54	0.45

1) The contents of crude protein in the ingredients.

2) Supplied per kg diet: Mn 75 mg, Zn 40 mg, Fe 80 mg, Cu 10 mg, iodine 0.3 mg, selenium 0.2 mg, retinol acetate 24 mg, DL-tocopheryl acetate 20 mg, cholecalciferol 0.034 mg, menadione 1 mg, thiamine 1.1 mg, riboflavin 3 mg, folic acid 1.2 mg, calcium pantothenate 5.5 mg, nicotinamide 30 mg, pyridoxine 2 mg, cobalamin 0.015 mg, biotin 0.2 mg.

3) Calculated value.

4) Analysed value.

**Table 2:** Sequences of the primers used for quantitative RT-PCR analysis

Genes	Forward	Reverse	References/Genebank No.
*AMPK*	AAGGTTGGCAAGCATGAGTT	TTCTGGGCCTGCATATAACC	Yi et al [13]
*PGC-1α*	CCAAAGGACACGCTCTAGATCA	TCTCGATCGGGAATATGGAGAA	Yi et al [13]
*ATP5B*	CGGCGGTTATTCGGTGTT	CCGTAGACCAGAGCGACCTT	NM_001031391
*HIF-1α*	CACCATTACCATACTTCAGCAG	CTTCACATCATCCACACGTTC	Osselaere et al [26]
*HMOX*	CTTGGCACAAGGAGTGTTAAC	CATCCTGCTTGTCCTCTCAC	Osselaere et al [26]
*XOR*	GTGTCGGTGTACAGGATACAGAC	CCTTACTATGACAGCATCCAGTG	Osselaere et al [26]
*GAPDH*	TGAAAGTCGGAGTCAACGGATT	CCACTTGGACTTTGCCAGAGA	Yi et al [13]

RT-PCR, quantitative real-time polymerase chain reaction; AMPK, AMP-activated protein kinase; PGC-1α, peroxisome proliferator-activated receptor coactivator-1α; ATP5B, ATP synthetase beta subunit; HIF-1α, hypoxia-inducible factor-1α; HMOX, heme-oxigenase; XOR, xanthine oxidoreductase; GAPDH, glyceraldehyde-3-phosphate dehydrogenase.

**Table 3:** Effects of NAC on growth performance of cold-stressed broilers

Items	Thermoneutral		Cold stress		SEM	p-value		
		
	Basal diet	NAC	Basal diet	NAC		Temperature	Diet	Interaction
d 1–14								
ADFI (g/d)	33.4	33.4	-	-	0.219	-	0.984	-
ADG (g/d)	22.9	22.6	-	-	0.223	-	0.562	-
FCR	1.46	1.48	-	-	0.008	-	0.302	-
d 15–42								
ADFI (g/d)	128	130	125	120	1.914	0.004	0.284	0.093
ADG (g/d)	67.1	68.3	58.5	56.1	1.395	<0.001	0.659	0.208
FCR	1.91	1.90	2.14	2.14	0.032	<0.001	0.757	0.951
Final BW (kg)	2.25	2.28	2.01	1.93	0.039	<0.001	0.580	0.188

NAC, N-acetylcysteine; SEM, standard error of the mean; ADFI, average daily feed intake; ADG, average daily gain; FCR, feed conversion ratio; BW, body weight.

**Table 4:** Effects of NAC on the relative weights of organs in cold-stressed broilers

Items	Thermoneutral		Cold stress		SEM	p-value		
		
	Basal diet	NAC	Basal diet	NAC		Temperature	Diet	Interaction
Heart (%)	0.61	0.67	0.84	0.81	0.036	<0.001	0.635	0.202
Liver (%)	2.02	1.95	2.11	2.33	0.111	0.040	0.514	0.205
Lung (%)	0.55	0.59	0.59	0.62	0.043	0.401	0.490	0.866
Thymus (%)	0.44^a^	0.21^b^	0.27^b^	0.26^b^	0.026	0.019	<0.001	<0.001
Spleen (%)	0.12	0.13	0.12	0.13	0.009	0.589	0.213	0.780
Bursa (%)	0.10^a^	0.07^ab^	0.05^b^	0.07^ab^	0.006	<0.001	0.513	0.011

NAC, N-acetylcysteine; SEM, standard error of the mean.

a,b Means with different letters in the same row differ significantly at p<0.05.

**Table 5:** Effects of NAC of plasma biochemical parameters of cold-stressed broilers

Items	Thermoneutral		Cold stress		SEM	p-value		
		
	Basal diet	NAC	Basal diet	NAC		Temperature	Diet	Interaction
ALT (U/L)	2.49	1.85	1.99	1.94	0.232	0.367	0.157	0.226
AST (U/L)	365	314	320	299	21.6	0.286	0.190	0.436
ALP (U/L)	1,653	1,157	2,459	1,813	144	<0.001	0.002	0.652
TP (g/L)	33.1	36.2	34.6	35.9	0.924	0.397	0.056	0.177
BUN (mmol/L)	0.88	0.84	0.80	0.78	0.031	0.202	0.754	0.937

NAC, N-acetylcysteine; SEM, standard error of the mean; ALT, alanine transaminase; AST, aspartate transaminase; ALP; alkaline phosphatase; TP, total protein; BUN, blood urea nitrogen.

**Table 6:** Effects of NAC on the concentrations of plasma amino acids in cold-stressed broilers

Amino acids (nmol/mL)	Thermoneutral		Cold stress		SEM	p-value		
		
	Basal diet	NAC	Basal diet	NAC		Temperature	Diet	Interaction
Essential amino acids								
Arginine	275	281	278	288	9.45	0.795	0.698	0.905
Glycine	557	560	544	573	12.6	0.993	0.553	0.638
Histidine	71.7	63.5	63.5	54.3	2.56	0.086	0.086	0.909
Isoleucine	76.7	58.7	88.6	88.4	3.18	<0.001	0.066	0.073
Leucine	135	121	146	143	4.35	0.066	0.319	0.548
Lysine	379	364	487	444	14.7	0.001	0.239	0.570
Methionine	52.6	50.9	61.4	61.2	1.97	0.016	0.787	0.845
Phenylalanine	94.8	99.8	95.7	97.1	1.85	0.818	0.405	0.644
Serine	688	672	602	583	17.7	0.014	0.603	0.952
Threonine	347	397	424	369	13.8	0.376	0.927	0.061
Valine	164	139	208	197	7.42	<0.001	0.117	0.549
Nonessential amino acids								
Alanine	905	804	657	673	25.7	<0.001	0.265	0.127
Asparagine	72.8^ab^	88.8^a^	87.3^a^	61.4^b^	3.44	0.282	0.410	0.001
Aspartate	54.3^ab^	42.5^b^^c^	38.6^c^	57.4^a^	2.30	0.912	0.361	<0.001
Cystine	49.4	56.5	55.3	61.6	1.59	0.071	0.030	0.885
Cysteine	2.60	3.18	2.96	4.14	0.19	0.117	0.034	0.588
Glutamate	142	131	131	139	5.36	0.867	0.900	0.413
Glutamine	857	859	741	785	18.2	0.008	0.491	0.532
Proline	204	192	175	187	4.32	0.043	0.972	0.151
Hydroxyproline	132	128	93.9	94.9	5.12	<0.001	0.836	0.749
Tyrosine	184	169	187	175	6.81	0.743	0.329	0.924
Tryptophan	28.7	29.3	10.5	16.6	1.68	<0.001	0.058	0.109
3-Methylhistidine	22.7	20.2	19.9	16.9	0.87	0.088	0.106	0.888

NAC, N-acetylcysteine; SEM, standard error of the mean.

a,b Means with different letters in the same row differ significantly at p<0.05.

**Table 7:** Effects of NAC on energy status in heart and liver of cold-stressed broilers

Items	Thermoneutral		Cold stress		SEM	p-value		
		
	Basal diet	NAC	Basal diet	NAC		Temperature	Diet	Interaction
Heart								
ATP (μg/g)	463	415	316	379	36.5	0.016	0.821	0.128
ADP (μg/g)	575	557	528	582	34.2	0.738	0.598	0.297
AMP (μg/g)	629	629	554	518	24.1	0.001	0.483	0.482
AMP/ATP	1.53	1.62	1.80	1.43	0.177	0.796	0.415	0.190
TAN1) (μg/g)	1,668	1,601	1,398	1,478	70.3	0.008	0.894	0.285
AEC2)	0.45	0.43	0.41	0.45	0.017	0.736	0.479	0.086
Liver								
ATP (μg/g)	54.9^a^	29.2^b^	34.8^b^	31.0^b^	3.52	0.014	<0.001	0.004
ADP (μg/g)	111^b^	173^a^	148.6^a^	157^a^	9.41	0.251	0.001	0.008
AMP (μg/g)	630	626	697	698	24.7	0.009	0.947	0.921
AMP/ATP	12.9	22.1	21.9	24.9	2.46	0.022	0.020	0.216
TAN1) (μg/g)	796	828	880	886	28.3	0.018	0.506	0.641
AEC2)	0.14	0.14	0.13	0.12	0.007	0.024	0.754	0.875

NAC, N-acetylcysteine; SEM, standard error of the mean; ATP, adenosine triphosphate; ADP, adenosine diphosphate; AMP, adenosine monophosphate; TAN, total adenine nucleotide; AEC, adenylate energycharges.

1) TAN = ATP+ADP+AMP.

2) AEC = (ATP+0.5 ADP)/TAN.

a,b Means with different letters in the same row differ significantly at p<0.05.

**Table 8:** Effects of NAC on redox status of cold-stressed broilers

Items	Thermoneutral		Cold stress		SEM	p-value		
		
	Basal diet	NAC	Basal diet	NAC		Temperature	Diet	Interaction
Plasma								
T-SOD (U/mL)	379	373	348	378	8.76	0.166	0.215	0.076
CAT (U/mL)	2.49	2.45	1.94	1.25	0.182	<0.001	0.071	0.148
GSH-Px (U/mL)	2211	2433	2264	2741	116	0.130	0.005	0.281
MDA (nmol/mL)	5.46^a^	3.41^b^	5.57^a^	5.64^a^	2.53	0.022	0.048	0.037
Heart								
T-SOD (U/mg prot)	189^a^	152^b^	153^b^	177^b^	7.68	0.564	0.788	<0.001
CAT (U/mg prot)	1.66	1.79	0.87	0.82	0.132	<0.001	0.756	0.494
GSH-Px (U/mg prot)	85.0	85.3	56.7	64.1	5.53	<0.001	0.528	0.559
MDA (nmol/mg prot)	0.62	0.66	0.79	0.84	0.914	0.030	0.404	0.894
Liver								
T-SOD (U/mg prot)	282^b^	237^c^	250^b^^c^	354^a^	10.2	<0.001	0.008	<0.001
CAT (U/mg prot)	4.27^a^	4.07^a^	2.60^b^	4.16^a^	0.284	0.014	0.026	0.043
GSH-Px (U/mg prot)	83.6^ab^	84.3^ab^	73.1^b^	95.7^a^	4.01	0.919	0.007	0.011
MDA (nmol/mg prot)	0.69	0.54	0.76	0.62	0.873	0.095	0.008	0.986

NAC, N-acetylcysteine; SEM, standard error of the mean; T-SOD, total superoxide dismutase; CAT, catalase; GSH-Px, glutathione peroxidase; MDA, malonaldehyde.

a,b Means with different letters in the same row differ significantly at p<0.05.
